# Countermovement Jump Performance Is Related to Ankle Flexibility and Knee Extensors Torque in Female Adolescent Volleyball Athletes

**DOI:** 10.3390/jfmk8020076

**Published:** 2023-06-07

**Authors:** Vassilios Panoutsakopoulos, Eleni Bassa

**Affiliations:** 1 Biomechanics Laboratory, School of Physical Education and Sport Science at Thessaloniki, Aristotle University of Thessaloniki, 54124 Thessaloniki, Greece; bpanouts@phed.auth.gr; 2 Laboratory of Evaluation of Human Biological Performance, School of Physical Education and Sport Science at Thessaloniki, Aristotle University of Thessaloniki, 54124 Thessaloniki, Greece

**Keywords:** biomechanics, sports performance, vertical jumps, isokinetics, stretch–shortening cycle, range of motion, ankle dorsiflexion, power, laterality, pubescent

## Abstract

Ankle flexibility and isokinetic knee torque/power generating capacity were previously suggested to contribute or to be correlated to the vertical countermovement jump (CMJ) performance. The aim of this study was to investigate the effect of the passive ankle joint dorsi flexion (θ_PDF_) and the knee muscle’s isokinetic torque and power on the CMJ in adolescent female volleyball players. The θ_PDF_ at a knee extension angle of 140 degrees were measured for 37 female post-pubertal volleyball players. Then, the players were assigned to either the flexible (*n* = 10) or inflexible (*n* = 14) groups according to earlier recommended criteria. Testing included the CMJ with and without an arm swing, and maximal knee extensions and flexions in 3 angular velocities on an isokinetic dynamometer. CMJ height performed with or without an arm swing (*r_(22)_* = 0.563, *p* = 0.040 and *r_(22)_* = 0.518, *p* = 0.009, respectively) and relative power (*r_(22)_* = 0.517, *p* = 0.010 and *r_(22)_* = 0.446, *p* = 0.030, respectively) were positively correlated with the extensors’ torque at 180°/s and were negatively correlated with the flexibility level of the dominant side ankle (*r_(22)_* = −0.529, *p* = 0.008 and *r_(22)_* = −0.576, *p* = 0.030, respectively). A moderate positive correlation was also revealed between the CMJ height with and without an arm swing and the power of the non-dominant knee extensors (*r_(22)_* = 0.458, *p* = 0.024 and *r_(22)_* = 0.402, *p* = 0.049, respectively) and flexors (*r_(22)_* = 0.484, *p* = 0.016 and *r_(22)_* = 0.477, *p* = 0.018, respectively). Results of the 2 × 2 repeated ANOVA measurements revealed that flexible players jumped significantly (*p* < 0.05) higher during the CMJs, whilst there was a group effect only on the isokinetic knee extensor muscles’ torque. In conclusion, a more flexible ankle joint and a higher isokinetic knee extensor’s torque generating capacity resulted in higher CMJ performance. Therefore, ankle flexibility should be emphasized in training and is suggested to be included in preseason screening tests of youth female volleyball players.

## 1. Introduction

Volleyball is a team sport, characterized by intermittent periods of high-intensity activities, interspersed by recovery periods of low intensity activities [1]. An increased jumping ability is considered a crucial fitness component for volleyball high level performance as point-scoring actions are mainly jump-based [2]. In addition, the vertical jump ability is highly associated with the attack action’s success [3].

A volleyball player’s jumping performance is the most assessed through squat jump (SJ), countermovement (CMJ), or/and drop jump (DJ) [4,5]. The SJ evaluates the jumping ability with only a concentric muscle action, whilst CMJ and DJ involve the utilization of the stretch–shortening cycle [6]. During vertical jumps, volleyball players mainly use a full arm swing with the arms initially swinging backward and then moving forward [7]. A coordinated arm swing shortens the braking phase and prolongs the accelerating phase of the jump [7], resulting in an enhanced jumping height and power output [8].

Due to the specificity of the volleyball game demands, it is imperative for players to possess not only coordination but also appropriate levels of strength and power [9]. A previous simulation training study using a musculoskeletal model of the major muscle groups contributing to jumping performance suggested the knee extensors’ training as the most effective means for improving it [10], whilst vastii muscles were proven to be great contributors to vertical center of mass (COM) acceleration during a CMJ [11]. Furthermore, high associations of muscular knee isokinetic peak torque and power with a vertical jump’s power and height were previously reported, although the kinematics of these two assessments (open vs. closed-chain activity) differ [12]. However, isokinetic knee testing is a common athlete’s evaluation that enables the determination of asymmetries (a) between the dominant and non-dominant limb—via the calculation of inter-limb torque deficit [13]—and (b) between knee flexors and extensors muscles via the conventional ratio, which, according to recent review [14], should be performed during both the preseason and in-season period for the better screening of deficits through a training macrocycle.

Performing a vertical jump requires mechanical energy generated by the proximal muscles to be transferred to a distal joint during the impulse. This energy transfer is facilitated by the bi-articular lower extremity muscles’ function [15] and flows from the hip to the knee and finally through the ankle joint, which contributes (~23%) via its plantar flexion to the take-off velocity [16]. The level of contribution of the ankle joint is dependent upon the torque generating capacity of the ankle plantar flexors with the bi-articular gastrocnemius muscle facilitating the energy flow because of the lag in its stimulation onset times [17]. An ankle joint’s range of motion and limited passive ankle dorsi flexion, in particular, is considered to be an important factor affecting jumping performance, as more flexible individuals outperform those of poor flexibility in jumping scores [18,19]. It was previously reported that ankle muscles’ strength may be determined by the range of motion [20]. Therefore, improving an ankle’s range of motion could possibly enhance jumping performance [21] by concurrently diminishing the possibility of injury. This is of importance for volleyball players, as ankle sprains are among the most common injuries they experience [22].

Reduced ankle mobility previously resulted in an impaired jumping performance in SJ in female adolescent volleyball players [23], suggesting the ankle range of motion as a crucial mediator of concentric-only jumping performance in the certain sport and age-group of female athletes. However, CMJ may be considered a more functional test for assessing vertical jumping performance in volleyball players, as it involves the use of the stretch–shortening cycle (SSC) and is highly associated with the spike jump performed during a volleyball match in both attacking- and serving-jump actions [3]. Despite the fact that inter-limb asymmetry is not evident in the bilateral CMJ [24], asymmetry in a single-leg CMJ is negatively related with performance in jumping and sprinting tests in youth team-sport athletes [25,26], with single-leg vertical jumps presenting larger asymmetries compared to horizontal jumping [27]. In line with these findings, volleyball players were found to exhibit a 13.6% greater single leg CMJ performance for the dominant leg [28]. This fact can be attributed to the different mechanical loading in each leg during spike jumps, which may alter the ankle range of motion as well.

Taking into consideration that vertical jumping [29] and isokinetic knee torque [30,31] evaluations are among the most common routine strength and conditioning assessments for volleyball players, it would be of great interest to examine the effect of ankle flexibility of both dominant and non-dominant leg, and knee torque generating capacity on CMJ performance in female adolescent volleyball athletes. We hypothesized that CMJ performance would be positively influenced by both of these previously mentioned variables.

## 2. Materials and Methods

### 2.1. Design of the Study

To fulfill the purpose of the study, after the assessment of the flexibility of the ankle joint, measures of the knee extensors and flexors isokinetic torque, and the examination of the biomechanical parameters of the CMJ with and without an arm swing were performed in random order.

### 2.2. Participants

Thirty-seven (*n* = 37) female pubescent volleyball players (16.5 ± 1.2 yrs, 1.80 ± 0.05 m, 68.5 ± 6.6 kg), selected to join the youth national teams, participated in this study. All participants were pubescent according to the Tanner [32] stages (Tanner stage V). This was also confirmed calculating the maturity offset [33], which was 4.19 ± 0.76 yrs for the inflexible (NFG) and 5.11 ± 0.84 yrs for flexible (FLX) group. Thus, all athletes were characterized as post-PHV. The players participated systematically in their training program (10–12 h/wk), had no injury for a 6-month period prior their evaluation and they were tested at least 24 h after the last strenuous training session. Their laboratory evaluation was a part of a wider physical conditioning screening program. The assessments were conducted in accordance with the Declaration of Helsinki and the Research Ethics Code of the Aristotle University of Thessaloniki.

### 2.3. Experimental Procedure

Firstly, the anthropometric characteristics of the participants were assessed. Body mass was measured to the nearest 0.1 kg using a digital weight scale (BC-545N, Tanita, Tokyo, Japan). A wall-mounted stadiometer (HR001; Tanita Tokyo, Japan) was used to assess the barefoot standing height to the nearest 0.1 cm. The dominant side was defined based on the preferred striking arm during the volleyball spike [34].

#### 2.3.1. Flexibility Assessment

Before the warm-up, the ankle joint flexibility test was conducted in a random order concerning the ipsilateral (DM) and contralateral (NDM) ankle joint of the dominant side. The passive non-weightbearing ankle joint dorsi flexion (θ_PDF_) [35], when the knee joint was fully extended (180° = full extension) and at a 140° angle, was measured. However, only the θ_PDF_ scores assessed at a 140° knee angle were further used, as this angle is suggested to consist of the representative lower limb configuration that is similar to the knee angle for vertical jumping execution in the majority of sports [36], and in volleyball sport-specific jumps, in particular [37].

Following this recommendation, θ_PDF_ was measured using a video analysis method [23]. A Panasonic NV-MS4E (Matsushita Electric Industrial Company, Osaka, Japan) camera (sampling frequency: 25 fps) was placed on a tripod (height: 1.2 m) at a distance of 4 m perpendicular to an examination bed. Before the measurement, the recorded field of view was calibrated using a 1.25 m × 1.25 m calibration frame with 10 reference markers. The participants sat and were fixed barefoot at the edge of the examination bed, with a hip angle of 120° [18], and the knee joint being at the edge of the bed at a 140° angle. Custom markers (diameter: 0.01 m) were attached on the tuberosity of the 5th metatarsal, the lateral malleolus, the posterior aspect of the calcaneus, the lateral epicondyle of the femur, and the greater trochanter.

For the measurement of θ_PDF_, force was applied from an experienced examiner on the plantar surface of the foot to dorsi-flex the ankle joint until a feeling of discomfort was stated by the participants [18]. Afterwards, the captured dorsi-flexion was projected on a COMPLOT 7000 digitizer (Mayline Company Inc., Sheboygan, WI, USA) after attaching the camera with a Citizen 30PC-1EB 1EA projector (Japan CBM Corp., Tokyo, Japan). The extracted coordinates of the digitized anatomical points in a two-dimensional Cartesian coordinate system were used to confirm the 140° knee joint angle and to compute θ_PDF_ with a 2D-DLT analysis method provided by the ANGLES 2004 software (©: Iraklis A. Kollias, Biomechanics Laboratory, Aristotle University of Thessaloniki, Thessaloniki, Greece).

The outcome of the θ_PDF_ measurement led to the formation of 2 experimental groups: the FLX (*n* = 10, 17.1 ± 0.9 yrs, 1.80 ± 0.04 m, 68.8 ± 5.7 kg), and the NFG (*n* = 14, 15.7 ± 0.8 yrs, 1.79 ± 0.05 m, 69.0 ± 7.3 kg) group. The cut-off thresholds for FLX and NFG were θ_PDF_ < 61° and θ_PDF_ > 69°, respectively. These cut-offs are suggested [38] to classify individuals as FLX (7.5th percentile) or NFG (92.5th percentile), respectively, based on the frequency distribution analysis of the Laboratory’s database that is comprised of a large cohort of female athletes and physical education students (*n* > 400). Inclusion in either group was considered only if the inter-limb difference for θ_PDF_ was less than 10°.

#### 2.3.2. Warm Up

A warm-up session followed the measurement of θ_PDF_. The players cycled for 8 min on an 817E Monark Exercise Cycle (Monark-Crescent AB, Varberg, Sweden). Then, they executed dynamic stretches with a progressively increasing range of motion. Finally, six CMJ, both without (CMJA) and with (CMJF) an arm swing and with increasing intensity from sub-maximum to maximum, were allowed for familiarization with the testing procedure.

#### 2.3.3. Vertical Jumps

Both CMJA and CMJF were performed on an AMTI OR6-5-1 force-plate (AMTI, Newton, MA, USA; sampling frequency: 1 kHz). All participants performed, in a random order, three CMJAs with arms kept akimbo and three CMJFs. The command was to “jump as fast and as high as possible”. No specific instruction was provided concerning the knee flexion during the countermovement. The intra-jump interval was 60 s, and the inter-test rest was 3 min.

Force-plate data acquisition and analysis was conducted using the routines of the K-Dynami 2018 (©: Iraklis A. Kollias, Biomechanics Laboratory, Aristotle University of Thessaloniki, Thessaloniki, Greece) software. The recorded vertical ground reaction force (vGRF) data was smoothed with a 2nd-order digital low pass Butterworth recursive filter. The cut-off frequency was set using the sum of residuals method [39] to 20 Hz. The jump height (H_CMJ_) was calculated from the COM vertical take-off velocity (V_0_) that was calculated as the first-time integral of the net vGRF using the trapezoid rule. The spatio-temporal (downward vertical COM displacement-S_D_; upward vertical COM displacement-S_U_; impulse time-t_C_; duration of the propulsion phase-t_PROP_) and the kinetic (net vGRF-Fz; rate of force development-RFD; peak power-P_MAX_) parameters of the CMJ tests were extracted based on the vGRF-time series, the participants’ mass, and classical equations of motion, as described in detail elsewhere [8]. The arm swing gain was estimated as the percentage chance of H_CMJ_ in CMJF compared to CMJA. The reactive strength index (RSI) was calculated as H_CMJ_/t_C_ [40], which is suggested to be an appropriate performance indicator for volleyball players [41]. For further analysis, only the best attempt, as defined by H_CMJ_, was selected.

#### 2.3.4. Isokinetic Evaluation

Participants performed concentric contractions of knee extensors and flexors seated (hip angle: 115°) on an isokinetic dynamometer (Cybex Norm, CYBEX Division of Lumex, Ronkonkoma, NY, USA). The trunk, waist, and upper thigh were stabilized on the chair using velcro straps to avoid any movement that could impact the measurement quality. Each participant raised the leg in parallel to the ground, correction of gravity was applied, full knee joint extension was checked, and the most prominent point of the medial femoral epicondyle was aligned with the axis of rotation of the dynamometer. Prior to the isokinetic test, participants performed 5 submaximal concentric knee flexions and extensions as familiarization. The isokinetic evaluation included 3 maximal knee extension and flexion trials at the concentric angular velocities of 60°/s and 180°/s performed in a randomized order. Knee range of motion was limited for all subjects from 0° to 90° of their knee flexion. The participants watched their torque scores on the screen of the dynamometer in order to outperform each previous trial (visual feedback) and were encouraged to perform their best in both movement directions. Inter-set rest was 3 min to avoid any fatigue effects. The highest peak torque and power values assessed at each angular velocity for both knee extensors and flexors were used for further analysis.

### 2.4. Statistical Analysis

According to the calculations using the G*power software (G*power, v.3.1.9.6, ©Franz Faul, University of Kiel, Kiel, Germany), the final sample size of 24 athletes used was the sample required for the present experimental design and it corresponded to 0.7 power for a 0.22 effect size at *a* = 0.05. The sample size was calculated based on the results of Panoutsakopoulos et al. [23].

All examined parameters were presented as mean ± standard deviation. The Shapiro–Wilk test (*p* > 0.05) and the Levene test (*p* > 0.05) were used to check the normality of distribution and the equality of variance, respectively. A 2 (flexibility; FLX, NFG) × 2 (arm swing: CMJA, CMJF) repeated-measures ANOVA with the Bonferroni adjustment was carried out to compare the main effects of flexibility and arm swing, and the interaction effect between flexibility and arm swing on the kinetic and temporal parameters of the CMJ. A 2 (angular velocity; 60°/s, 180°/s) × 2 (groups: FLX, NFG) repeated-measures ANOVA with the Bonferroni adjustment was carried out to compare the main effect of the angular velocity and group and the interaction effect between angular velocity × group on the torque and power of knee extensors and flexors and the conventional ratio of DM and NDM limbs. An Independent Samples *t*-test was run to check possible group differences in the CMJ gain due to the arm swing. Hedges’ *g* was used to interpret the effect size of the comparison. A Pearson correlation coefficient (*r*) was computed to assess the linear relationship between the H_CMJ_ in CMJA and CMJF and the knee flexors’ and extensors’ torque and power.

The statistical analyses were conducted with the IBM SPSS Statistics v.27.0.1.0 software (International Business Machines Corp., Armonk, NY, USA). The level of significance was set at *a* = 0.05 for all analyses.

## 3. Results

### 3.1. Passive Ankle Dorsi Flexion

**Figure 1:**
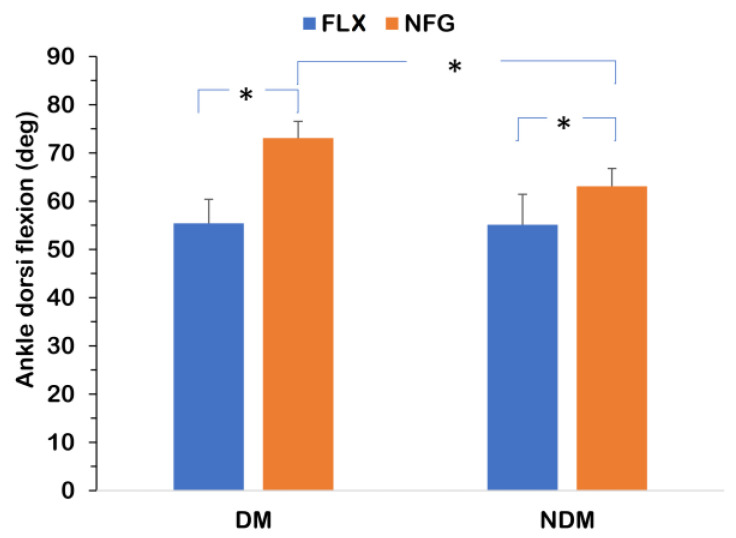
Results of the passive ankle dorsi flexion measurement (FLX: flexible group, *n* = 10; NFG: inflexible group, *n* = 14; DM: ipsilateral ankle joint of the preferred arm for the spike; NDM: contralateral ankle joint of the preferred arm for the spike; *: *p* < 0.05).

The results of the flexibility measurements are depicted in Figure 1. A significant effect of laterality on θ_PDF_ (*F_(1,22)_* = 38.89, *p* < 0.001, *η_p_^2^* = 0.64), a significant group effect (FLX > NFG; *F_(1,22)_* = 57.01, *p* < 0.001, *η_p_^2^* = 0.72), and an interaction of laterality × group (*F_(1,22)_* = 34.49, *p* < 0.001, *η_p_^2^* = 0.61) was found. The FLX players presented no significant inter-limb difference (*p* > 0.05), whilst the NFG had a lower θ_PDF_ in the DM leg (*p* < 0.001).

### 3.2. Countermovement Jumps

#### 3.2.1. CMJ Height

A significant effect of the arm swing on H_CMJ_ was found (*F_(1,22)_* = 187.52, *p* < 0.001, *η_p_^2^* = 0.90). A significant difference between groups in H_CMJ_ in both conditions (no arm swing and arm swing) was detected, with FLX players presenting higher scores (*F_(1,22)_* = 17.23, *p* < 0.001, *η_p_^2^* = 0.44). No significant (*p* > 0.05) interaction of the arm swing × group was found (Table 1).

#### 3.2.2. CMJ Arm Swing Gain

No significant difference was observed for the gain in CMJ due to the arm swing (*t_(1,23)_* = 0.20, *p* = 0.843, *g* = 0.09). It was 17.7 ± 5.0% for the FLX and 18.3 ± 8.2% for the NFG.

#### 3.2.3. CMJ Biomechanics

No significant effect of arm swing on the CMJ peak net vGRF relative to body mass was found (*p >* 0.05) but a significant group effect was detected (*F_(1,22)_* = 7.73, *p* < 0.01, *η_p_^2^* = 0.26), since FLX presented higher values than NFG (Table 1). A significant effect of arm swing on the CMJ relative power (*F_(1,22)_* = 88.70, *p* < 0.001, *η_p_^2^* = 0.80) and RSI (*F_(1,22)_* = 6.74, *p* = 0.017, *^η^_p_^2^*= 0.23) was found.

A significance between the groups’ difference in CMJ relative power and RSI in both conditions (no arm swing and swing) was detected, with the more flexible athletes presenting higher power (*F_(1,22)_* = 19.62, *p* < 0.001, *η_p_^2^* = 0.47) and RSI (*F_(1,22)_* = 19.03, *p* < 0.001, *η_p_^2^* = 0.46) values. Concerning the maximum RFD, as well as S_D_, S_U_, t_C_, and t_PROP_, no significant effect of the arm swing (*p >* 0.05) and no significant group effect (*p >* 0.05) was found. Finally, no significant interaction of the arm swing × group was found for all of the above-mentioned parameters (*p* > 0.05).

### 3.3. Isokinetic Tests

#### 3.3.1. Isokinetic Torque

An angular velocity effect was found for the knee flexors’ torque of the DM (*F_(1,22)_* = 184.64, *p* < 0.001, *η_p_^2^* = 0.89) and NDM (*F_(1,22)_* = 306.24, *p* < 0.001, *η_p_^2^* = 0.93) lower limbs (Table 2). Neither a significance between the groups’ difference in the dominant knee flexors’ torque nor an interaction of the angular velocity × group was found (*p* > 0.05).

An angular velocity effect on the knee extensors’ torque of the DM (*F_(1,22)_* = 443.69, *p* < 0.001, *η_p_^2^* = 0.95) and NDM (*F_(1,22)_* = 244.25, *p* < 0.001, *η_p_^2^* = 0.92) leg was found. A significant between the groups’ difference in the knee extensors’ torque of both lower limbs was found (*F_(1,22)_* = 5.394, *p* < 0.030, *η_p_^2^* = 0.20 and *F_(1,22)_* = 7.413, *p* < 0.012, *η_p_^2^* = 0.25 for DM and NDM, respectively), with FLX presenting a higher knee extensors’ torque. No significant interaction of the angular velocity × group was found (*p* > 0.05).

#### 3.3.2. Inter-Limb Torque Deficit

No effect of the muscle group on inter-limb deficit was found, no difference between the groups was detected, and no interaction between the muscle groups × group difference was found (*p* > 0.05).

#### 3.3.3. Conventional Ratio

An angular velocity effect on the conventional ratio of the DM and NDM leg was found, with the ratio being higher at 60°/s (*F_(1,22)_* = 16.85, *p* < 0.001, *η_p_^2^* = 0.43 and *F_(1,22)_* = 16.85, *p* < 0.001, *η_p_^2^* = 0.43 for DM and NDM, respectively). No difference between the groups and no interaction of the angular velocity × group was found (*p* > 0.05).

#### 3.3.4. Isokinetic Power

An angular velocity effect on the knee flexors’ power of the DM (*F_(1,22)_* = 63.28, *p* < 0.001, *η_p_^2^* = 0.74) and NDM (*F_(1,22)_* = 65.52, *p* < 0.001, *η_p_^2^* = 0.75) leg was found (Table 3), as knee flexors’ power presented higher values at 180°/s compared to 60°/s. In addition, an angular velocity effect on the knee extensors’ power of the DM (*F_(1,22)_* = 194.37, *p* < 0.001, *η_p_^2^* = 0.90) and the NDM (*F_(1,22)_* = 305.48, *p* < 0.001, *η_p_^2^* = 0.93) leg was observed, with the knee extensors’ power presenting higher values at 180°/s compared to 60°/s. Neither a significance between the groups’ difference in DM and NDM knee flexors’ power, nor a significance between the groups’ difference in DM and NDM knee extensors’ power was found (*p* > 0.05). Finally, no interaction of the angular velocity × group on isokinetic power was revealed (*p* > 0.05).

### 3.4. Correlations

There was a significant positive moderate correlation between the CMJA and CMJF H_CMJ_ and the extensors’ torque of the DM leg at 180°/s (*r_(22)_* = 0.563, *p* = 0.040 and *r_(22)_* = 0.518, *p* = 0.009, respectively). There was also a significant positive moderate correlation between the CMJA and CMJF H_CMJ_ and the NDM leg extensors’ torque at 180°/s (*r_(22)_* = 0.514, *p* = 0.010 and *r_(22)_* = 0.456, *p* = 0.025, respectively).

A significant positive moderate correlation between the CMJA and CMJF and the flexors’ power of the NDM leg at 180°/s was observed (*r_(22)_* = 0.484, *p* = 0.016 and *r_(22)_* = 0.477, *p* = 0.018, respectively). In addition, a significant positive moderate correlation between the CMJA and CMJF and the NDM extensors’ power at 180°/s was revealed (*r_(22)_* = 0.458, *p* = 0.024 and *r_(22)_* = 0.402, *p* = 0.049, respectively).

A significant moderate correlation between the DM leg extensors’ torque at 180°/s and the CMJA and CMJF relative power was detected (*r_(22)_* = 0.517, *p* = 0.010 and *r_(22)_* = 0.446, *p* = 0.030, respectively). This was also observed for the NDM leg, since a significant moderate correlation between the NDM leg extensors’ torque at 180°/s and the CMJA and CMJF relative power was detected (*r_(22)_* = 0.461, *p* = 0.020 and *r_(22_*_)_ = 0.414, *p* = 0.040, respectively).

A significant negative moderate correlation between the CMJA and CMJF and the θ_PDF_ of the DM leg was detected (*r_(22)_* = −0.529, *p* = 0.008 and *r_(22)_* = −0.576, *p* = 0.030, respectively). Similarly, a significant negative correlation between the CMJA and CMJF P_MAX_ and the θ_PDF_ of the DM leg was found (*r_(22)_* = −0.535, *p* = 0.007 and *r_(22)_* = −0.586, *p* = 0.003, respectively).

No other significant correlations were revealed.

## 4. Discussion

The current study examined the hypothesis that the countermovement jump performance with and without arm swing in female pubescent volleyball players could be affected by (a) ankle flexibility, (b) by asymmetries in ankle flexibility between ipsilateral and contralateral to spike arm leg, and/or by (c) knee flexors’ and extensors’ torque. The hypothesis was confirmed as the FLX players, who presented no inter-limb difference in the passive ankle joint dorsi flexion, jumped higher than the less flexible, who presented a relatively restricted passive ankle joint dorsi flexion at the ipsilateral to spike arm leg. Regarding isokinetic knee torque, the NFG produced lower knee extensors’ torque than FLX in both 60°/s and 180°/s. Furthermore, the CMJ height and power output in the trials performed with or without an arm swing were positively correlated with the extensors’ torque at 180°/s and were negatively correlated with the flexibility level of DM ankle, suggesting that both parameters are significant mediators of CMJ performance.

The FLX jumped higher in the CMJ than NFG, corroborating previous results in SJ, where ankle flexibility affected the SJ performance in female volleyball players [23]. This could be attributed to the larger force and power output [8,42,43,44,45]. In the less flexible or reduced ankle mobility conditions, the reduced contribution of the biarticular gastrocnemius to the energy transfer was previously reported to be counterbalanced with the larger mobility of the torso and the hip joint [36], and an augmented knee mechanical output [46]. The present findings revealed that FLX also showed larger knee extensors’ torque compared to the NFG. This indicates the poor capacity of the latter to both produce [47] and transfer energy for the jump. This factor was found not to change in female volleyball players during adolescence [48], but is suggested to discriminate between skilled and players of lesser abilities during this age [49]. Taking this into consideration, one would expect that NFG—although they have a flexibility deficit—to have adapted through the great number of sport-specific jumps performed during volleyball training and they would have probably found an optimal way to perform the CMJs. Although kinematic analysis was beyond the aims of this study, the lower jumping height, force, velocity, and power values in NFG athletes suggested that this was probably not the case.

RSI comprises of a temporal normalization of jump height, categorizing jumping activities in slow or fast. FLX presented higher RSI scores than NFG, proving not only their ability to jump higher than NFG, but also the ability to jump faster, which is considered the desirable way to perform jumps in athletic activities. Therefore, we may also assume that the FLX athletes probably store and release more energy, demonstrating superior and more effective utilization of the SSC [50] during CMJ than their inflexible counterparts. RSI is considered a sensitive indicator of efficient neuromuscular function and lower limb explosiveness in female volleyball athletes [51]. Furthermore, RSI was previously highly correlated with higher force, power, velocity, and impulse during jumps [52]—parameters, in which, FLXs were found to have larger values than the NFGs.

As mentioned above, the parameters interpreting explosiveness in vertical jumps, such as power and reactive strength, were augmented with the arm swing and a flexible ankle joint. The examined groups were different in the force output in the no-arm swing CMJ, but not in the CMJ with an arm swing. The arm swing generates mechanical work that is transferred and imposes a greater load to the lower limb muscles, thus leading to a higher capacity to produce energy for the jump [42,53]. Thus, it seems that the inflexible players used the additional work produced by the arm swing to limit the deficiency in energy production due to the limited mobility of the ankle joint. However, the energy transfer from the upper to the lower limbs should be sequentially synchronized throughout the jump [53]. Past research [18] utilizing kinematical analysis of the CMJ revealed differences between flexible and inflexible individuals in the body configuration and the rotational kinematics of the lower limb joints, concluding that inflexible individuals absorb energy during the eccentric phase of the CMJ that is not compensated during the propulsion phase, thus leading to a decreased jumping performance due to limited movement amplitudes [54]. The lack of a kinematical analysis deprives the extraction of solid evidence concerning this mechanism.

Interestingly, there was a significant interaction of laterality and flexibility with only the NFG presenting significant differences in flexibility between the dominant and non-dominant leg. Laterality differences in the flexibility may be attributed to the different sport-specific demands of jumps for the joint kinematics of each leg in volleyball [9,37]. Volleyball athletes perform a lot of spike jumps that load differently the ipsi- and contra-lateral to spike arm leg. Variations in weight distribution, power development, ankle angle at foot planting, and pressure experienced at ipsi- and contra-lateral leg during foot planting, are different neuromuscular stimuli that could result in specific adaptations [34]. Generally speaking, skeletal muscle tissue may remodel its structure adapting to mechanical loading [55]. Thus, higher forces applied during the sport-specific jumps on the contralateral to spike arm leg may have probably resulted in a higher ankle range of motion compared to the ipsilateral leg in the NFG. This assumption could be further supported by the notion that the higher the force accelerating the ankle motion, the greater the ankle range of motion [56]. Previous research in handball players revealed that laterality, based on hand preference, resulted in significant differences in the active ankle joint range of motion at the selected knee angle where the flexibility test in the present study was conducted [57]. Additionally, a recent study applying a stretching protocol to 1 leg for 12 weeks reported increases in the ankle range of motion of the non-trained leg, a finding that led the researchers speculate that volleyball training, per se, probably affects ankle flexibility [58]. However, such kind of adaptations were not apparent in the FLX, suggesting that a flexible joint probably remains at its level of flexibility in both legs, regardless of the inter-limb loading differences. Furthermore, flexibility differences between groups may also be attributed to an age effect, as the NFG players were younger than the FLX (15.66 ± 0.77 yrs vs. 17.05 ± 0.94 yrs, respectively). This finding further supports the hypothesis that flexibility may be altered during pubescence by specific sport training [58], since muscles and joints probably have the potential to adapt, while growing, to meet a particular sport-specific performance requirement [59].

Warm-up before any athletic action is considered essential to optimize performance [60]. In detail, the warm-up procedure elevates temperature, which may decrease the viscosity of the tissues [60], resulting in a lower resistance to stretch and an increased joint range of motion [61]. However, in the present study, no warm-up was performed prior the flexibility measurements, because warm-up was found to have no effect on flexibility compared to the control condition (i.e., no warm-up), whilst any increases in flexibility were observed only after stretching [62]. Finally, the present study design is further supported by similar research in the literature, where warm-up was performed after the flexibility testing session and before the execution of the jumping tests [18].

We are aware that our research may have limitations. The isokinetic evaluation was conducted only in the knee joint as this assessment of the knee extensor and flexor muscles is a common practice in preseason athletes’ screening [63]. An additional ankle torque evaluation would probably offer better insight in the probable contribution of ankle muscles in the CMJ performance in female volleyball athletes of different ankle flexibility. Furthermore, a kinematic analysis could provide additional information about the differences between flexible and inflexible young female volleyball players in terms of posture and knee joint rotational kinematics. Future research using kinematic recordings and isokinetic evaluation of ankle joint muscles needs to be conducted. Finally, asymmetry is affected by the training season [27]. The fact that flexibility asymmetry was assessed in a single day during the end-season may have affected not so much the magnitude, but rather the direction of asymmetry [64], probably resulting in different bilateral CMJ scores, compared to scores assessed during other training seasons, a case that would be interesting to be examined as well.

## 5. Conclusions

Vertical jump performance is crucial for high level performance in volleyball. According to the findings of this study, female adolescent volleyball players who present a high ankle joint flexibility and can exert high isokinetic knee extensors’ torque outperform their less flexible and weaker counterparts in the vertical jump performance. Therefore, to improve the vertical jumping ability of female adolescent volleyball athletes throughout their long-term training, coaches are encouraged not only to check their athletes for knee muscles’ torque deficits, but also for any possible ankle flexibility deficits or asymmetries through different training seasons, and to train them accordingly in order to augment performance and concurrently limit injury occurrence possibility. As adolescence is an important time for laying the foundations for long-term athletic development, an individual approach, in both flexibility and strength development, is suggested for maximizing training effects whilst supporting sound physical development.

## Figures and Tables

**Table 1:** Parameters for the countermovement jump without (CMJA) and with (CMJF) the use of an arm swing in the flexible (FLX, *n* = 10) and non-flexible (NFG, *n* = 14) groups.

Parameter	Test	FLX (*n* = 10) (Mean ± SD)	NFG (*n* = 14) (Mean ± SD)	Flexibility		Arm Swing		Interaction	
				*p*	*η_p_^2^*	*p*	*η_p_^2^*	*p*	*η_p_^2^*
H_CMJ_ (cm)	CMJA CMJF	25.22 ± 3.25 29.74 ± 4.29 ^#^	20.21 ± 2.77 * 23.81 ± 2.89 *^#^	<0.001	0.44	<0.001	0.90	0.13	0.10
V_0_ (m/s)	CMJA CMJF	2.22 ± 0.15 2.41 ± 0.19 ^#^	2.00 ± 0.14 * 2.16 ± 0.13 *^#^	<0.001	0.42	<0.001	0.90	0.43	0.03
FZmax (N/kg)	CMJA CMJF	2.43 ± 0.21 2.42 ± 0.18	2.21 ± 0.25 * 2.31 ± 0.16	0.01	0.26	0.48	0.02	0.38	0.04
RFDmax (kN/s)	CMJA CMJF	10.40 ± 4.10 7.55 ± 2.01 ^#^	8.43 ± 4.22 8.64 ± 3.55	0.72	0.01	0.14	0.10	0.90	0.12
P_MAX_ (W/kg)	CMJA CMJF	24.63 ± 2.83 31.22 ± 4.74 ^#^	19.31 ± 2.75 * 25.72 ± 3.20 *^#^	<0.001	0.47	<0.001	0.80	0.90	0.001
S_D_ (cm)	CMJA CMJF	−30.01 ± 5.12 −29.95 ± 4.11	−31.01 ± 3.79 −29.12 ± 4.83	0.96	0.00	0.18	0.08	0.37	0.04
S_U_ (cm)	CMJA CMJF	50.91 ± 6.05 53.87 ± 4.25	48.94 ± 4.37 49.52 ± 5.49	0.07	0.14	<0.001	0.57	0.58	0.02
t_C_ (ms)	CMJA CMJF	597.60 ± 126.74 651.50 ± 141.72	638.36 ± 101.55 642.64 ± 131.12	0.59	0.01	0.50	0.02	0.57	0.02
t_PROP_ (ms)	CMJA CMJF	306.20 ± 36.19 334.70 ± 56.92	332.00 ± 37.66 338.21 ± 45.85	0.23	0.06	0.23	0.07	0.43	0.03
RSI (m/s)	CMJA CMJF	0.83 ± 0.12 0.92 ± 0.22	0.62 ± 0.11 0.72 ± 0.12	<0.001	0.46	0.017	0.23	0.92	0.002

NOTE: H_CMJ_: jump height; V_0_: body center of mass vertical take-off velocity; FZmax: peak net vertical ground reaction force; RFDmax: peak rate of force development; P_MAX_: peak power; S_D_: downward center of mass vertical displacement; S_U_: upward center of mass vertical displacement; t_C_: duration of the impulse; t_PROP_: duration of the propulsive phase; RSI: reactive strength index; *: *p* < 0.05 vs. FLX; ^#^: *p* < 0.05 vs. CMJA.

**Table 2:** Isokinetic torque values of knee extensors (Ext) and flexors (Flex) at 60°/s and 180°/s for the dominant (DM) and the non-dominant (NDM) leg in the flexible (FLX, *n* = 10) and non-flexible (NFG, *n* = 14) groups.

Laterality	Torque (Nm)	FLX (*n* = 10) (Mean ± SD)	NFG (*n* = 14) (Mean ± SD)	Group		Angular Velocity		Interaction	
				*p*	*η_p_^2^*	*p*	*η_p_^2^*	*p*	*η_p_^2^*
DM	Ext 60°/s Ext 180°/s	186.10 ± 25.77 129.50 ± 20.04 ^#^	163.71 ± 25.03 * 111.50 ± 16.12 *^#^	0.03	0.20	<0.001	0.95	0.41	0.03
NDM	Ext 60°/s Ext 180°/s	181.20 ± 27.50 123.80 ± 17.43 ^#^	154.21 ± 20.61 * 108.36 ± 15.98 *^#^	0.01	0.25	<0.001	0.92	0.10	0.01
DM	Flex 60°/s Flex 180°/s	98.90 ± 17.79 60.60 ± 17.49 ^#^	92.50 ± 17.43 57.00 ± 13.49 ^#^	0.43	0.03	<0.001	0.89	0.61	0.01
NDM	Flex 60°/s Flex 180°/s	94.40 ± 21.21 59.00 ± 17.99 ^#^	90.64 ± 16.56 57.00 ± 14.54 ^#^	0.68	0.01	<0.001	0.93	0.66	0.01

NOTE: Ext 60°/s: torque of knee extensors at 60°/s; Ext 180°/s: torque of knee extensors at 180°/s; Flex 60°/s: torque of knee flexors at 60°/s; Flex 180°/s: torque of knee flexors at 180°/s; *: *p* < 0.05 vs. FLX; ^#^: *p* < 0.05 vs. 60°/s.

**Table 3:** Isokinetic power values of knee extensors (Ext) and flexors (Flex) at 60°/s and 180°/s for the dominant (DM) and the non-dominant (NDM) leg in the flexible (FLX, *n* = 10) and non-flexible (NFG, *n* = 14) groups.

Laterality	Power (W)	FLX (*n* = 10) (Mean ± SD)	NFG (*n* = 14) (Mean ± SD)	Group		Angular Velocity		Interaction	
				*p*	*η_p_^2^*	*p*	*η_p_^2^*	*p*	*η_p_^2^*
DM	Ext 60°/s Ext 180°/s	111.46 ± 27.21 207.55 ± 65.22 ^#^	112.86 ± 19.62 209.64 ± 31.26 ^#^	0.90	0.001	<0.001	0.90	0.96	0.00
NDM	Ext 60°/s Ext 180°/s	117.90 ± 21.39 230.70 ± 46.03 ^#^	112.06 ± 16.64 204.19 ± 34.35 ^#^	0.17	0.08	<0.001	0.93	0.09	0.12
DM	Flex 60°/s Flex 180°/s	66.40 ± 10.90 104.08 ± 31.11 ^#^	64.04 ± 10.97 103.17 ± 26.26 ^#^	0.83	0.002	<0.001	0.74	0.88	0.00
NDM	Flex 60°/s Flex 180°/s	68.31 ± 20.52 1119.63 ± 56.45 ^#^	60.50 ± 10.35 101.91 ± 22.76 ^#^	0.27	0.06	<0.001	0.75	0.40	0.03

NOTE: Ext 60°/s: power of knee extensors at 60°/s; Ext 180°/s: power of knee extensors at 180°/s; Flex 60°/s: power of knee flexors at 60°/s; Flex 180°/s: power of knee flexors at 180°/s; ^#^: *p* < 0.05 vs. 60°/s.

## Data Availability

The data that were used in the present study can be provided by the corresponding author upon reasonable request.
